# Relationship between incident types and impact on patients in drug name errors: a correlational study

**DOI:** 10.1186/s40780-015-0011-x

**Published:** 2015-03-10

**Authors:** Toshikazu Tsuji, Toshihiro Irisa, Shunichi Ohata, Chiyo Kokubu, Akiko Kanaya, Masanori Sueyasu, Nobuaki Egashira, Satohiro Masuda

**Affiliations:** Department of Pharmacy, Kyushu University Hospital, 3-1-1 Maidashi, Higashi-ku, Fukuoka, 812-8582 Japan

**Keywords:** Drug name errors, Preparation errors, Inspection errors, Incidents, Impact on patients

## Abstract

**Background:**

There are many reports regarding various medical institutions’ attempts at incident prevention, but the relationship between incident types and impact on patients in drug name errors has not been studied. Therefore, we analyzed the relationship between them, while also assessing the relationship between preparation and inspection errors. Furthermore, the present study aimed to clarify the incident types that lead to severe patient damage.

**Methods:**

The investigation object in this study was restricted to “drug name errors”, preparation and inspection errors in them were classified into three categories (similarity of drug efficacy, similarity of drug name, similarity of drug appearance) or two groups (drug efficacy similarity (+) group, drug efficacy similarity (−) group). Then, the relationship between preparation and inspection errors was investigated in three categories, the relationship between incident types and impact on patients was examined in two groups.

**Results:**

The frequency of preparation errors was liable to be caused by the following order: similarity of drug efficacy > similarity of drug name > similarity of drug appearance. In contrast, the rate of inspection errors was liable to be caused by the following order: similarity of drug efficacy < similarity of drug name < similarity of drug appearance. In addition, the number of preparation errors in the drug efficacy similarity (−) group was fewer than that in the drug efficacy similarity (+) group. However, the rate of inspection errors in the drug efficacy similarity (−) group was significantly higher than that in the drug efficacy similarity (+) group. Furthermore, the occupancy rate of preparation errors, incidents more than Level 0, 1, and 2 in the drug efficacy similarity (−) group increased gradually according to the rise of patient damage.

**Conclusions:**

Our results suggest that preparation errors caused by the similarity of drug appearance and/or drug name are likely to lead to the incidents (inspection errors), and these incidents are likely to cause severe damage to patients subsequently.

**Electronic supplementary material:**

The online version of this article (doi:10.1186/s40780-015-0011-x) contains supplementary material, which is available to authorized users.

## Background

Medical care security measures have advanced, but medical accidents and incidents continue to occur. Needless to say, pharmacists should make every effort to prevent incidents caused by their own errors. We have been working on countermeasures for preventing incidents regarding oral and external drugs in the pharmaceutical department of Kyushu University Hospital, and we have been able to maintain the occurrence rate of incidents of these drugs in the range from 0.027 to 0.036% for seven years, ever since April 2007 [1-3]. However, it is very difficult for pharmacists to prevent all incidents in actuality.

In general, it is recognized that incidents involved in drug name errors can easily cause profound damage to patients in terms of problem severity, but it is not clear what impact the occurrence of these incidents might have on patients subsequently. There are many reports regarding the prevention of incidents in oral and external drugs in various medical institutions [1-5], and many analytical studies have been conducted regarding the probability of drug name confusion [6-11], but the relationship between incident types and impact on patients in drug name errors has not been studied. In this study, preparation and inspection errors involved in drug name errors were classified into three categories (similarity of drug efficacy, similarity of drug name, similarity of drug appearance) or two groups (drug efficacy similarity (+) group, drug efficacy similarity (−) group). Then, the relationship between preparation and inspection errors was analyzed in three categories, the relationship between incident types and impact on patient was examined in two groups.

## Methods

### The investigation period and object

The investigation period lasted seven years, from April 2007 to March 2014. The investigation object was preparation and inspection errors amidst inpatient punctual prescriptions. Among them, the errors of narcotics, powders, injections, and tablets divided by an automatic packaging machine were excluded from this study because of the difference in dispensing procedures. Furthermore, the definitive investigation object in this study was restricted to “drug name errors”, not including “drug count errors”, “drug content errors” and so on.

Preparation errors were equivalent to dispensing the drugs incorrectly, and these data was self-reported by pharmacy inspectors. Also, inspection errors were equivalent to overlooking the preparation errors caused by dispensers, and these data was reported by other medical staff. In Kyushu University Hospital, prescriptions for dispensing are printed in the pharmaceutical department using the prescription operating system, and medicines in the pharmaceutical department are arranged in accordance with the classification of drug efficacy. In addition, the contents of prescriptions and the years of pharmacist’s experience were not analyzed in this study.

### Definition of incidents and classification of incident impact on patients

We defined the errors detected by other medical staff or inpatients after being overlooked by pharmacists or inspectors as “incidents.” According to the provisions of the National University Hospital in Japan, impact on patients of the incidents was classified into 6 stages (Levels 0–5) as described below.

- Level 0: Incorrect drug was delivered to other medical staff or patient, but it was not used.
- Level 1: Incorrect drug was used by a patient, but actual patient damage was not caused.
- Level 2: Moderate damage was caused to patient, but treatment was not needed.
- Level 3: Provisional or continual treatment was needed.
- Level 4: Severe damage to patient remained.
- Level 5: Patient died.

### Definition of preparation and inspection errors

We defined the errors detected by the pharmacy inspector as “preparation errors,” the errors not detected by pharmacy inspectors as “inspection errors.” In this study, it was considered the practical preparation errors to be equivalent to all errors, including incidents more than Level 0, because incidents more than Level 0 were simply not detected by the pharmacists at the point of inspection.

In short, the number of preparation errors contained all inspection errors, the number of inspection errors contained the incidents of Levels 0–5, and inspection errors had an equivalent meaning to “incidents more than Level 0.” The definition of preparation and inspection errors was summarized as described below.

- Preparation errors: The errors that were revealed to be incorrect afterward. They were equivalent to the “all errors” category, and these included errors detected by pharmacy inspector.
- Inspection errors: The errors that were not detected by pharmacy inspectors, and they are equivalent in meaning to “incidents more than Level 0”.

### Classification of preparation and inspection errors into three categories

Preparation and inspection errors involved in drug name errors were classified into four categories: (1) similarity of drug efficacy, (2) similarity of drug name, (3) similarity of appearance, and (4) no similarities. Also, the category of no similarities (4) was used as a comparison (control) group.

We defined the drugs having common efficacy in insurance adaptation as “similarity of drug efficacy”. Also, the trade names of Japanese drugs are expressed by *katakana* in most cases, and *katakana* expression in Japanese consists of both orthographic (i.e., spelling) and phonological (i.e., pronunciation) factors. Therefore, the trade name of the *katakana* was converted into a Romanized version of Japanese (non-English words Romanized using Hepburn’s method), because it represents the exact features of the *katakana*. Then, we defined the drugs having commonality of continuous letters in Romanized Japanese as “similarity of drug name”. Furthermore, we defined the drugs having similar colors, shapes, and/or sizes as “similarity of drug appearance”, they were evaluated by the both tablets/capsules and blister-packages.

In the process of the classification of these errors into three categories (1) - (3), the errors were able to be divided into seven detailed error types (a) - (g) as follows: (a) similarity of drug efficacy alone, (b) similarity of drug name alone, (c) similarity of drug appearance alone; three more, (d) similarity of drug efficacy and name, (e) similarity of drug efficacy and appearance, (f) similarity of drug name and appearance; and one, (g) similarity of drug efficacy, name, and appearance.

Also, we defined the errors that did not overlap with other similarities as “single similarity class: (a) + (b) + (c),” errors overlapped with two similarities as “double similarity class: (d) + (e) + (f)”, and errors overlapped with three similarities as “triple similarity class: (g).” Furthermore, the control group was regarded as “no similarity class”. Then, the differences in the rates of inspection errors were analyzed among four error classes (single, double, triple and no similarity class) and among three error types (a) - (c) in the single similarity class and another three error types (d) - (f) in the double similarity class. Additional file 1: Table S1 shows the classification method of categories (1) - (3) and the actual cases of preparation errors.

### Reclassification of preparation and inspection errors, and incident impact on patients in two groups

It was considered that impact on patients of the incidents involved in drug name errors would be affected by either the presence or absence of drug efficacy similarity. Therefore, we reclassified preparation errors, inspection errors, and the subsequent incident impact on patients into two groups: “drug efficacy similarity (+) group” and “drug efficacy similarity (−) group.” Then, the relationship between preparation and inspection errors was analyzed, and the relationship between incident types and their impact on patients was examined in two groups.

### Analysis of preparation and inspection errors

The differences in preparation errors among the error classes, types and groups were analyzed by the number of errors based on the number of total prescriptions over seven years, because each number indicates the frequency of occurrence on condition that total prescription number is invariable. On the other hand, the differences in inspection errors among them were analyzed by the rate of errors that calculated by dividing the number of inspection errors by that of preparation errors.

### Statistical analysis

The data were analyzed with a Chi-squared test and a Ryan test. P values of <0.05 were considered to be statistically significant. A Chi-squared test was used to analyze differences in the rates of inspection errors among four error classes (single, double, triple and no similarity class) and between two groups (drug efficacy similarity (+) group, drug efficacy similarity (−) group). Also, a Ryan test as multiple comparison was used to analyze differences in the rates of inspection errors among three error types (a) - (c) in the single similarity class and another three error types (d) - (f) in the double similarity class.

## Results

### Numbers of preparation and inspection errors

**Figure 1:**
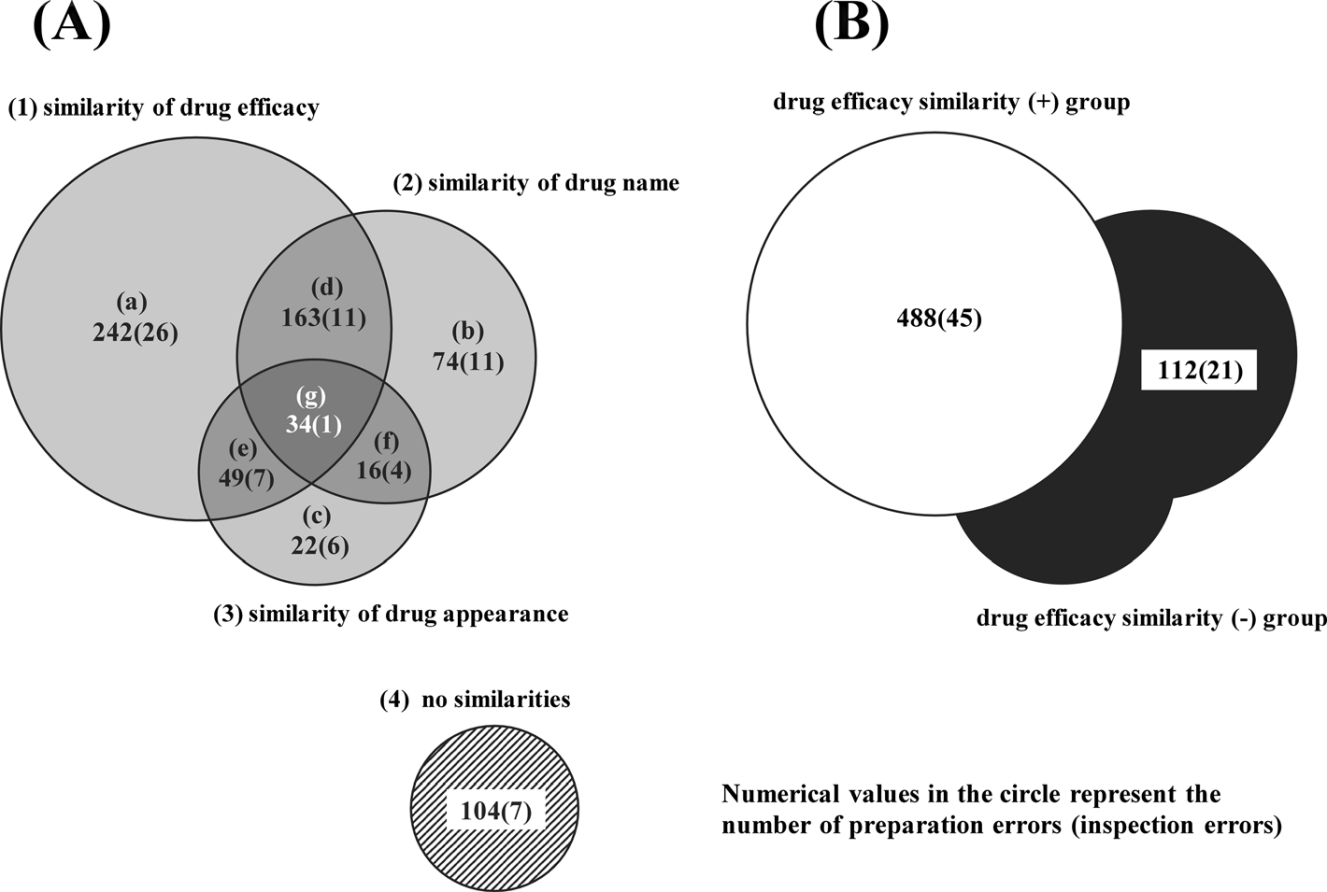
**Number of preparation and inspection errors in three categories (A) and two groups (B).** The total numbers of preparation and inspection errors involved in drug name errors were 704 and 73, respectively. Also, the numbers of preparation and inspection errors in three categories (similarity of drug efficacy, similarity of drug name, similarity of drug appearance) or two groups (drug efficacy similarity (+) group, drug efficacy similarity (−) group) were 600 and 66, respectively. The preparation and inspection errors were classified into seven error types **(A)** and two error groups **(B)**. Numerical values in the circle represent the number of preparation errors (inspection errors).

Over the seven years, 664,887 inpatient punctual prescriptions were given. The total numbers of preparation and inspection errors involved in drug name errors were 704 and 73, respectively. Also, the numbers of preparation and inspection errors in three categories (similarity of drug efficacy, similarity of drug name, similarity of drug appearance) or two groups (drug efficacy similarity (+) group, drug efficacy similarity (−) group) were 600 and 66, respectively. Figure 1 shows the number of preparation and inspection errors in three categories (A) and two groups (B).

### Relationship between number of preparation errors and rate of inspection errors in four classes

**Figure 2:**
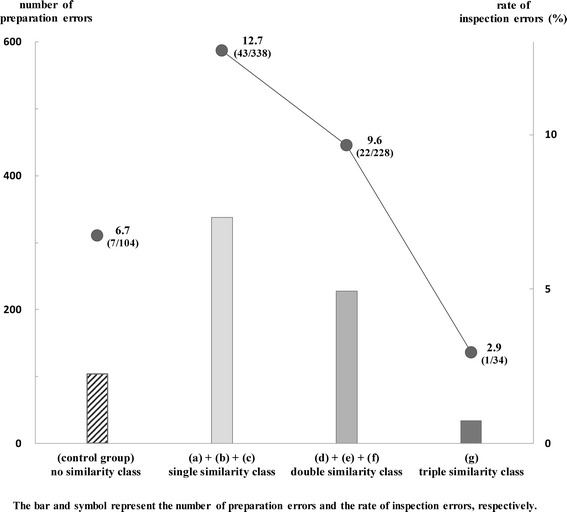
**Relationship between number of preparation errors and rate of inspection errors in four classes.** The numbers of preparation errors in three classes (single similarity class, double similarity class, triple similarity class) were 338, 228, and 34, the rates of inspection errors in the same classes were 12.7% (43/338), 9.6% (22/228), and 2.9% (1/34), respectively. The bar and symbol represent the number of preparation errors and the rate of inspection errors, respectively. The differences in the rates of inspection errors were analyzed among four error classes (single, double, triple and no similarity class). There were no significant differences in the rates of inspection errors among four classes (P = 0.1305). The data were analyzed with a Chi-squared test. P values of <0.05 were considered to be statistically significant.

Figure 2 shows the relationship between the number of preparation errors and the rate of inspection errors in four classes. The number of preparation errors and the rate of inspection errors in the control group (no similarity class) were 104 and 6.7% (7/104). Also, the numbers of preparation errors in three classes (single, double, and triple similarity class) were 338, 228, and 34, the rates of inspection errors in the same classes were 12.7% (43/338), 9.6% (22/228), and 2.9% (1/34), respectively.

There were no significant differences in the rates of inspection errors among four classes (P = 0.1305). Also, a positive correlation was observed between the numbers of preparation errors and the rates of inspection errors in three classes (single, double, and triple similarity class).

### Relationship between number of preparation errors and rate of inspection errors in the single and double similarity class

**Figure 3:**
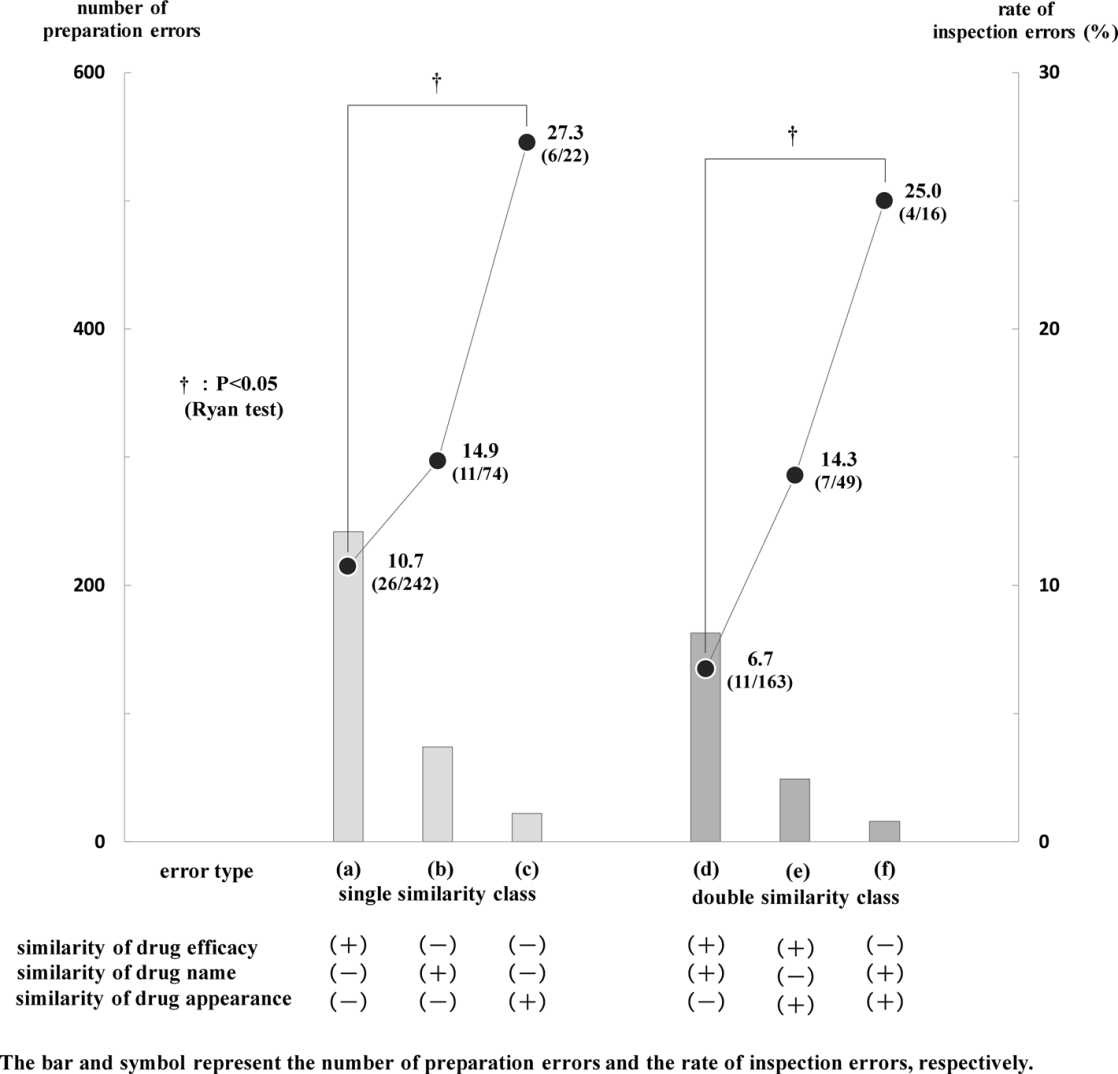
**Relationship between number of preparation errors and rate of inspection errors in the single and double similarity class.** In the single similarity class, the numbers of preparation errors in the error type **(a)**, **(b)**, **(c)** were 242, 74, 22, and the rates of inspection errors in the same error type were 10.7% (26/242), 14.9% (11/74), 27.3% (6/22), respectively. In the double similarity class, the numbers of preparation errors in error types **(d)**, **(e)**, **(f)** were 163, 49, 16, and the rates of inspection errors were 6.7% (11/163), 14.3% (7/49), 25.0% (4/16), respectively. The bar and symbol represent the number of preparation errors and the rate of inspection errors, respectively. The differences in the rates of inspection errors were analyzed among three error types **(a) - (c)** in the single similarity class and another three error types **(d) - (f)** in the double similarity class. There were significant differences in the rates of inspection errors between error type **(a) and (c)** in the single similarity class, between error type **(d) and (f)** in the double similarity class (P < 0.05). The data were analyzed with a Ryan test. P values of <0.05 were considered to be statistically significant.

Figure 3 shows the relationship between the number of preparation errors and the rate of inspection errors among three error types (a) - (c) in the single similarity class and another three error types (d) - (f) in the double similarity class. In the single similarity class, the numbers of preparation errors in the error type (a), (b), (c) were 242, 74, 22, and the rates of inspection errors in the same error type were 10.7% (26/242), 14.9% (11/74), 27.3% (6/22), respectively. In the double similarity class, the numbers of preparation errors in error types (d), (e), (f) were 163, 49, 16, and the rates of inspection errors were 6.7% (11/163), 14.3% (7/49), 25.0% (4/16), respectively.

There were significant differences in the rates of inspection errors between error type (a) and (c) in the single similarity class, between error type (d) and (f) in the double similarity class (P < 0.05). Also, an inverse correlation was observed between the numbers of preparation errors and the rates of inspection errors in both the single and double similarity classes.

Furthermore, when we compared the rates of inspection errors between error types (d) and (e) based on the common similarity (similarity of drug efficacy) for both, it was revealed that the rate of inspection errors having “similarity of drug appearance” was higher than that having “similarity of drug name”. By comparing the rates of inspection errors between error type (d) and (f), between error type (e) and (f) in the double similarity class, it was revealed comprehensively that the rate of inspection errors was liable to be caused by the following order: similarity of drug efficacy < similarity of drug name < similarity of drug appearance, while the frequency of preparation errors was liable to be a result of the reverse order.

### Relationship between number of preparation errors and rate of inspection errors in two groups

**Figure 4:**
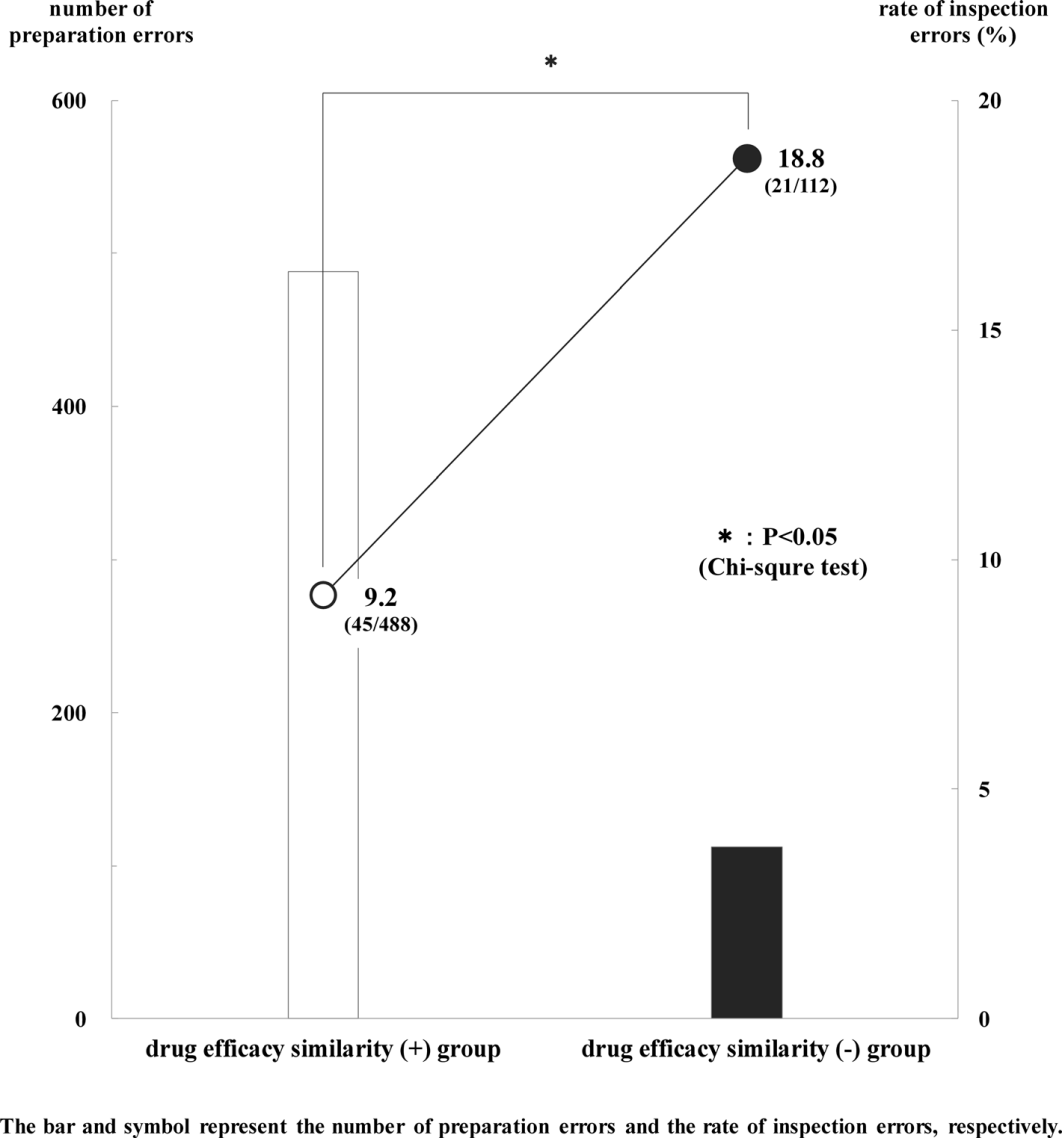
**Relationship between number of preparation errors and rate of inspection errors in two groups.** The number of preparation errors was 488, and the rate of inspection errors was 9.2% (45/488) in the drug efficacy similarity (+) group. The number of preparation errors was 112, and the rate of inspection errors was 18.8% (21/112) in the drug efficacy similarity (−) group. The bar and symbol represent the number of preparation errors and the rate of inspection errors, respectively. The differences in the rates of inspection errors were analyzed between two groups (drug efficacy similarity (+) group, drug efficacy similarity (−) group). There was a significant difference in the rates of inspection errors between two groups (P < 0.05). The data were analyzed with a Chi-squared test. P values of <0.05 were considered to be statistically significant.

Figure 4 shows the relationship between the number of preparation errors and the rate of inspection errors in two groups (drug efficacy similarity (+) group, drug efficacy similarity (−) group). In the drug efficacy similarity (+) group, the number of preparation errors was 488, and the rate of inspection errors was 9.2% (45/488). In the drug efficacy similarity (−) group, the number of preparation errors was 112, and the rate of inspection errors was 18.8% (21/112).

There was a significant difference in the rates of inspection errors between two groups (P < 0.05). Also, an inverse correlation was observed between the numbers of preparation errors and the rates of inspection errors in two groups.

### Relationship between incident types and impact on patients

**Figure 5:**
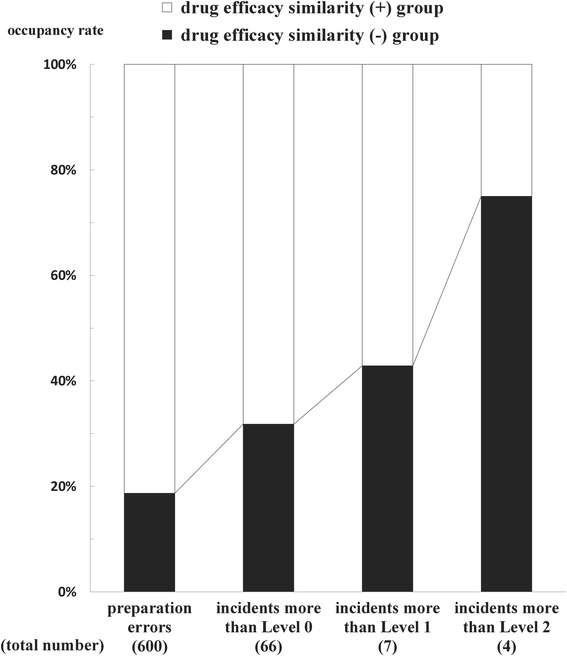
**Relationship between incident types and impact on patients.** The occupancy rates of preparation errors, incidents more than Level 0, 1, and 2 in the drug efficacy similarity (+) group were 81.3% (488/600), 68.2% (45/66), 57.1% (4/7), and 25.0% (1/4), respectively. On the other hand, the same rates in the drug efficacy similarity (−) group were 18.7% (112/600), 31.8% (21/66), 42.9% (3/7), and 75.0% (3/4), respectively. Consequently, the occupancy rate of preparation errors, incidents more than Level 0, 1, and 2 in the drug efficacy similarity (−) group increased gradually according to the rise of patient damage.

Figure 5 shows the relationship between incident types and their impacts on patients. There were no incidents more than Level 3. The occupancy rates of preparation errors, incidents more than Level 0, 1, and 2 in the drug efficacy similarity (+) group were 81.3% (488/600), 68.2% (45/66), 57.1% (4/7), and 25.0% (1/4), respectively. On the other hand, the same rates in the drug efficacy similarity (−) group were 18.7% (112/600), 31.8% (21/66), 42.9% (3/7), and 75.0% (3/4), respectively.

The usage rate of patients after delivering the incorrect medicines (more than Level 1/ more than Level 0) was 8.9% (4/45) in the drug efficacy similarity (+) group, while the same rate was 14.3% (3/21) in the drug efficacy similarity (−) group. Furthermore, the occurrence rate of moderate damage to patients after taking the incorrect medicines (more than Level 2/ more than Level 1) was 25.0% (1/4) in the drug efficacy similarity (+) group, while the same rate was 100% (3/3) in the drug efficacy similarity (−) group.

## Discussion

A positive correlation was observed between the numbers of preparation errors and the rates of inspection errors in three classes (single, double, and triple similarity class). On the other hand, an inverse correlation was observed between them in the single and double similarity class. In short, the frequency of preparation errors is liable to be caused by the following order: similarity of drug efficacy > similarity of drug name > similarity of drug appearance, while the rate of inspection errors is liable to be caused by the following order: similarity of drug efficacy < similarity of drug name < similarity of drug appearance. These results suggest that preparation and inspection errors differ entirely in terms of their occurrence factors. Pharmacists are likely to dispense inappropriate drugs resembling the correct drug in their efficacy, because pharmaceutical department medicines are arranged in accordance with the classification of drug efficacy. On the other hand, pharmacy inspectors are likely to overlook the preparation errors caused by the similarity of drug appearance and/or name, because they tend to make judgments based on the color, shape, and size of a drug, or based on parts of letters in a drug’s name.

Interestingly, the number of preparation errors in the drug efficacy similarity (−) group was fewer than that in the drug efficacy similarity (+) group, while the rate of inspection errors in the drug efficacy similarity (−) group was significantly higher than that in the drug efficacy similarity (+) group. Furthermore, the occupancy rate of preparation errors, incidents more than Level 0, 1, and 2 in the drug efficacy similarity (−) group increased gradually according to the rise of patient damage. These results suggest that preparation errors caused by the similarity of drug appearance and/or name (preparation errors not caused by the similarity of drug efficacy) are liable to lead to the incidents (inspection errors), furthermore, these incidents are liable to cause the severe patient damage subsequently.

For these reasons, pharmacy inspectors need to take steps to prevent such inspection errors. As a countermeasure for them, it would be useful for pharmacy inspectors to confirm additionally the “identification code” indicated on the exterior of each medicine. For example, the identification codes corresponding to medicines are supposed to be indicated on the prescription for dispensing by enrolling them at our hospital pharmacy. Therefore, it is possible for us to compare a pair of the same codes on both exterior of medicine and prescription for dispensing. Because the identification code is typically a simple and unique mark with the combination of number, symbol, and so on, it is unlikely for pharmacy inspectors to be influenced by preconceptions in terms of comparing the two codes.

## Conclusions

In conclusion, the preparation errors caused by the similarity of drug appearance and/or drug name are likely to lead to the incidents (inspection errors). Furthermore, these incidents are likely to cause severe damage to patients subsequently. It is important for pharmacists to utilize these results for preventive measure of errors and for pharmaceutical education.

## Additional file

Additional file 1: Table S1. Classification method into (1) - (3) categories and actual cases of preparation errors.
