# Supplementary material for: Relationship between incident types and impact on patients in drug name errors: a correlational study
Source: J Pharm Health Care Sci. 2015 Mar 10;1:11. doi: 10.1186/s40780-015-0011-x (PMC4729157; doi:10.1186/s40780-015-0011-x)
Supplement: Additional file 1: Table S1. — Classification method into (1) - (3) categories and actual cases of preparation errors. [file 40780_2015_11_MOESM1_ESM.pdf]

Table 1 Classification method into (1) - (3) categories and actual cases of preparation errors

Error category	Classification method
**(1) Similarity of drug efficacy**	**Both of correct drug and incorrect drug have the common efficacy in insurance adaptation.**
**(2) Similarity of drug name**	**Trade names of both drugs according to Romanized Japanese satisfy one or more items in the following four items**

1. Three or more letters from beginning are the same
2. Four or more letters from ending are the same
3. Two letters from beginning or three letters from ending are the same, and the numerical value of drug content is the same, too
4. Besides the above three items, both drug names have the commonality of five or more successive letters.

※ The letters expressed singly as “A”, “I”, “U”, “E”, “O”, “N” and the letters of “SHI”, “CHI”, “TSU” are defined as “two letters” exceptionally.

Error category	Classification method
**(3) Similarity of drug appearance**	**Appearances of both drugs satisfy two or more items in the following three items**

1. Colors of both drugs (tablets/capsules) are similar, and colors of both drug blister-packages are similar, too
2. Shapes of both drug blister-packages are similar, or arrangements of both drugs (tablets/capsules) are similar
3. Sizes of both drug blister-packages are similar (area ratio $\geqq (0.9)^2 = 0.81$, volume ratio $\geqq (0.9)^3 = 0.73$)

	**Single similarity class**	**Double similarity class**	**Triple similarity class**
**(1) Similarity of drug efficacy**	**error type (a)**<br>**error cases of (1) alone**<br>Voltaren (25mg) ⇒ Loxoprofen (60mg)<br>Amoban (7.5mg) ⇒ Lendormin (0.25mg)	**error type (d)**<br>**error cases of (1) and (2)**<br>Alfarol (1μg) ⇒ Onealfa (0.5μg)<br>●A/RU/FUA/RO/O/RU (1μg) ⇒ WA/N/A/RU/FUA (0.5μg)<br>Constan (0.4mg) ⇒ Lexotan (1mg)<br>●KO/N/SU/TA/N (0.4mg) ⇒ RE/KI/SO/TA/N (1mg)	
**(2) Similarity of drug name**<br>**●Drug name in Romanized Japanese**	**error type (b)**<br>**error cases of (2) alone**<br>Mucosta (100mg) ⇒ Mucodyne (250mg)<br>●MU/KO/SU/TA (100mg) ⇒ MU/KO/DA/I/N (250mg)<br>Decadron (0.5mg) ⇒ Depas (0.5mg)<br>●DE/KA/DO/RO/N (0.5mg) ⇒ DE/PA/SU (0.5mg)	**error type (f)**<br>**error cases of (2) and (3)**<br>Calonal (200mg) ⇒ Caltan (500mg)<br>●KA/RO/NA/A/RU (200mg) ⇒ KA/RU/TA/N (500mg)<br>Caloryl Jelly (40.496%) ⇒ Argamate Jelly (20%)<br>●KA/RO/RI/I/RU ZE/RI/I (40.496%)<br>⇒ A/A/GA/ME/I/TO ZE/RI/I (20%)	**error type (g)**<br>**error cases of (1) and (2) and (3)**<br>Bio-Three (1g) ⇒ Biofermin-R (1g)<br>●BI/O/SU/RI/I (1g) ⇒ BI/O/FUE/RU/MI/N・【R】(1g)<br>Ensure Liquid (250ml) ⇒ Ensure-H (250ml)<br>●E/N/SHU/A LI/KI/DDO (250ml)<br>⇒ E/N/SHU/A・【H】(250ml)
**(3) Similarity of drug appearance**	**error type (c)**<br>**error cases of (3) alone**<br>Zyloric (100mg) ⇒ Calonal (200mg)<br>Lasix (40mg) ⇒ Warfarin (1mg)	**error type (e)**<br>**error cases of (3) and (1)**<br>Proheparum (combination) ⇒ EPL (250mg)<br>Bonalon (35mg) ⇒ Actonel (17.5mg)	

(1) Similarity of drug efficacy
error type (a)
error cases of (1) alone

The underline represents the common points between correct drug and incorrect drug.
